# Hepatic adenoma in a 7-year-old girl: a case report and literature review

**DOI:** 10.1186/s12887-023-04209-5

**Published:** 2023-08-24

**Authors:** Yan Gao, Jun Zhou, Yu-cheng Xie, Li-juan Qiu, Ling Duan, Zhi-xiang A, Hong-fang Wu, Meng-xing Lv

**Affiliations:** 1 Department of pathology, Kunming Children’s Hospital, 288 Qianxing Road, Kunming, 650028 Yunnan China; 2 Second People’s Hospital of Yunnan Province, 176 Qingnian Road, Kunming, 650034 Yunnan China

**Keywords:** Hepatocellular adenoma, Pediatric, Pathological presentation, Case report

## Abstract

**Background:**

Hepatocellular adenomas (HCAs) are rare benign tumors of the liver that occur predominantly in women taking oral contraceptives. In children, HCAs comprise < 5% of hepatic tumors. We report a case of HCAs in a 7-year-old girl with estrogen and glucose imbalance.

**Case presentation:**

A 7-year-old girl was presented to our hospital with bilateral breast enlargement for 2 months, polydipsia, polyuria, polyphagia, hyperglycemia, and significant weight gain. Computed tomography (CT) showed a 7.2 cm×6.9 cm×5.3 cm round-shaped mass in the left inner lobe of the liver, ovarian ultrasound showed multiple follicles in the ovaries bilaterally, and cranial magnetic resonance imaging (MRI) showed an enlarged superior pituitary. Hematological and biochemical results were as follows: fasting glucose was 19.7 mmol/L, estradiol was 122.9 pmol/L, follicle-stimulating hormone 10.81 IU/L, luteinizing hormone 10.99 IU/L, insulin-like growth factor 1,513 ng/mL, glutamine aminotransferase 86 U/L, and alkaline phosphatase 362 U/L. Thyroid functions, methemoglobin, fetal protein, carcinoembryonic antigen, and chorionic gonadotropin were normal. The patient had a complete surgical resection of the liver tumor, and the postoperative histopathological diagnosis was HCAs. After the surgery, insulin was injected and the glucose levels were stable. During the 36-month follow-up period, neither tumor recurrence nor significant abnormalities were detected using color Doppler ultrasound of the liver. The child’s precocious puberty is currently under control.

**Conclusions:**

HCAs are particularly rare in children with liver tumors, and risk factors for the development of HCAs in children include sex hormone imbalance, obesity, Fanconi anemia (FA), glycogen storage diseases (GSDs) type I, III, and IV, galactosemia, immunodeficiency, congenital portosystemic shunts (CPSS), cardiac hepatopathy status-post Fontan procedure, Hurler syndrome, familial adenomatous polyposis, germline *HNF1A* mutations, and maturity-onset diabetes of the young type 3. Most HCAs are detected during a physical examination without clinical symptoms, and some patients may present with symptoms such as abdominal pain, abdominal distension, and abdominal masse. Serum liver function tests can show increased alkaline phosphatase (ALP) and γ- glutamyl transferase (GT), whereas α-Fetoprofein (AFP) levels are normal. The definitive diagnosis relies mainly on histopathological examination. Because HCAs can rupture and bleed and become malignant. Early surgical treatment is recommended after detection.

## Background

Hepatocellular adenomas (HCAs) are rare benign tumor of the liver that occurs mainly in women taking oral contraceptives. The occurrence of HCAs in children is rare. In children, HCAs comprise less than 5% of hepatic tumors and are associated with various conditions [1]. Risk factors for the development of HCAs in children include sex hormone disorders, a variety of metabolic and immune defects, and mutations in *HNF1α* [2]. Here, we report a case of HCAs in children associated with estrogen and glucose imbalance.

## Case presentation

**Fig. 1:**
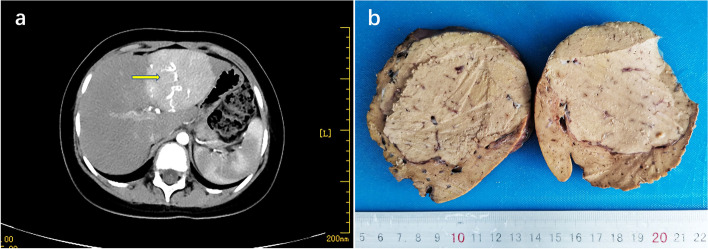
Computed tomography (CT) showed a round-shaped mass in the left inner lobe of the liver (arrowhead) (**a**). Gross specimen of the tumor (**b**)

**Fig. 2:**
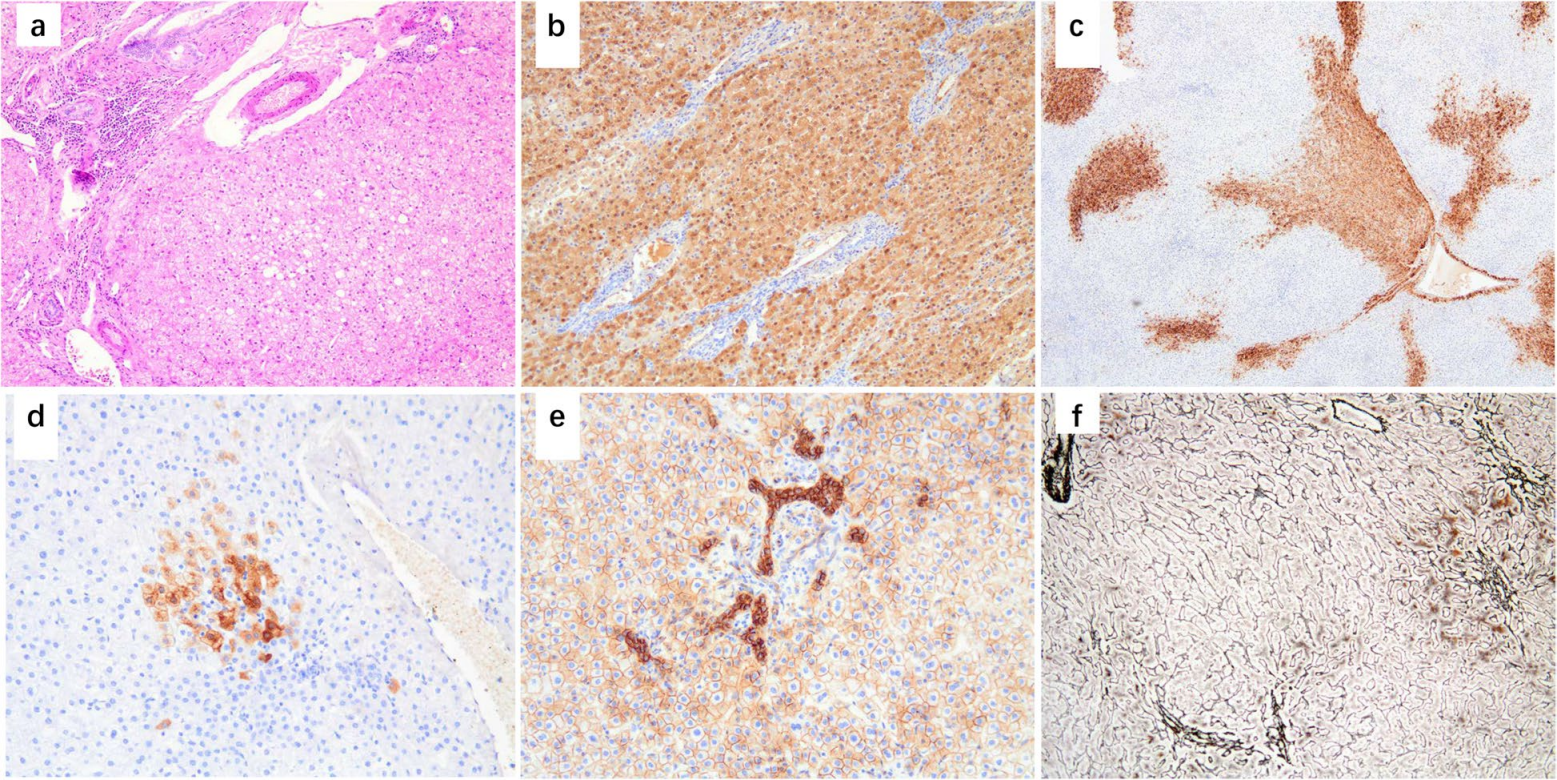
The hepatic lobular structure was not evident in the tumor area, and that there were no portal areas or central veins, tumor cells were similar to normal hepatocytes with translucent cytoplasm and slightly larger nuclei in a few cells, a small amount of lymphocytic infiltration around bile ducts (H&E, magnification x100) (**a**). Immunoreactive for L-FABP (magnification x100) (**b**). Immunoreactive for GS (magnification x40) (**c**). Immunoreactive for CRP (magnification x200) (**d**). Immunoreactive for β-catenin (positive expression on cell membrane, magnification x200) (**e**). Reticulin stain revealed disordered cell cord arrangement and slight hepatic plate thickening in some areas (magnification x100) (**f**)

The female patient (G2P1) was born by cesarean section via in vitro fertilization, with a birth weight of 3.35 kg. She resented to our hospital with bilateral breast enlargement for 2 months, polydipsia, polyuria, increased appetite, hyperglycemia, and significant weight gain. The patient did not take birth control pills or bee syrup. Fasting blood sugar level was19.7 mmol/L. During the examination, there was no rash over the whole body and no significant enlargement of the thyroid gland. Scattered café-au-lait spots on the left buttock and left inner thigh. Both breasts were stage B2, the vulva was not mature, and no pubic hair growth was observed. Computed tomography (CT) enhancement of the upper abdomen suggested a class of rounded reinforcing occupying foci with clear borders in the left lobe of the liver, which was approximately 5.85 cm×6.76 cm (anterior-posterior diameter × left-right diameter) in size, with uniform reinforcement of the occupying parenchyma and visible tortuous and thickened blood supply arteries within (Fig. 1a). A magnetic resonance imaging (MRI) scan of the pituitary gland showed a pituitary size of 6.7 cm×6.9 cm×10.4 mm (upper and lower diameter × anterior and posterior diameter × right and left diameter), which suggested an enlargement of the superior pituitary margin. Ovarian color doppler ultrasound showed multiple follicles in both ovaries. Hematological and biochemical results were as follows: fasting glucose 19.7 mmol/L (3.9–5.8), estradiol 122.9 pmol/L (22–99), follicle-stimulating hormone 10.81 IU/L (0.4–5), luteinizing hormone 10.99 IU/L (0.1–0.4), insulin-like growth factor 1 513 ng/mL (64–345), glutamine aminotransferase 86U/L (0–50), and alkaline phosphatase 362U/L (147.7–309.3). Thyroid function, fetoprotein, carcinoembryonic antigen, and chorionic gonadotropin were normal. The patient underwent tumor removal and pathological examination. Gross examination was as follows: one gray-red-yellow liver mass, size 9 cm×7.5 cm×4.5 cm, solid in section, with clear demarcation between tumor and liver tissue visible (Fig. 1b). Histological analysis revealed that the hepatic lobular structure was not evident in the tumor area, and that there were no portal areas or central veins. There were no hemorrhagic lesions or cystic changes were observed. The hepatocellular adenoma was composed of tumor cells of uniform size arranged in 1–2 layers of cellular cords, with compressed hepatic sinusoid spaces between the cords. The tumor cells were comparable in size to normal hepatocytes or slightly larger, with a normal nuclear/cytoplasmic ratio, the absence of mitotic figures and cell atypia, and transparent cytoplasm (Fig. 2a). Microscopically, the boundary between the tumor and contiguous liver tissue was unclear, the liver cells were edematous, the portal area and the portal area and central vein were present, and there was no evidence of bleeding, cystic degeneration, cirrhosis, or other pathological alterations. In some areas, the Reticulin stain revealed a disorganized arrangement of cell fibers and a modest thickening of the hepatic plate. IHC staining revealed L-FABP(+) expression (Fig. 2b), GS(partial+) (Fig. 2c), CRP(foci+) (Fig. 2d), SAA(foci+), β-catenin (positive on cell membrane, +) (Fig. 2e), AAT(+), CD34(foci+), and disordered configuration of cell fibers as revealed by the reticulin stain. In some locations, the hepatic plate was marginally thickened (Fig. 2f). The conclusive pathological diagnosis was of HCAs (subtype: unclassified HCA, U-HCA). The patient received insulin injections after the procedure and maintained stable glycemic control. The patients were observed for a duration of 36 months (as of 2023.07.10). The liver color Doppler ultrasound results revealed no recurrence of the tumor and no apparent abnormalities. The normal levels of follicle-stimulating hormone (FSH), luteinizing hormone (LH), estradiol (E2), progesterone (P), testosterone (T), and prolactin (PRL) indicate that the child’s precocious puberty is currently under control.

## Discussion and conclusions

HCAs are rare benign tumors caused by hepatocytes, occurring in 3–4 cases per 100,000 people [3]. HCAs are hormone-driven tumors. Tumor size is significantly correlated with hormone levels, and half of the tumors decrease or stop growing when oral hormonal drugs are stopped. Glycogen accumulation disorder type I, maturity-onset diabetes of the young type 3, familial adenomatous polyposis, hemoglobin deposition disorder, chronic alcoholism, obesity, and metabolic syndrome are also assumed to be associated with the occurrence of HCAs. Liu et al. [4] retrospectively analyzed the data of 189 patients with HCA in China and sequenced three signature genes of HCAs, including *HNF1α*, *β-catenin*, and *gp130*, in 36 patients with HCAs. All HCAs had mutations in *HNF1α*, and mutations in β-catenin and *gp130* was not been detected in HCAs. WHO (2010 classification) classifies HCAs into the following four subtypes based on genotype and clinical presentation: *hepatocyte nuclear factor 1 homeobox alpha (HNF1A)*-inactivated HCAs (HHCAs), inflammatory HCAs (IHCAs), *beta-catenin*-mutated HCAs (bHCAs), and unclassified HCAs (UHCAs) [5]. Further evaluation using RNA sequencing, whole-exome, and genome sequencing yielded extended classifications, including the following: bHCAs involving exon 3 (b^ex3^HCAs) and exon 7 or 8 (b^ex7,8^HCAs), ICHAs with *beta-catenin* mutations (b^ex3^IHCAs and be^x7,8^IHCAs), and a newly defined entity of sonic hedgehog HCAs (shHCAs) [6].

### Pathological characteristics

The majority of HCAs are well-defined single nodules, but in a few cases, there can be multiple nodules. Nodules more than > 10 are called adenomatosis. The masses range from 0.5 to 26 cm in diameter, with little or no fibrous envelope. The mass is grayish red in section, soft and uniform, and can show cholestasis, bruising and hemorrhage, clearly demarcated from the surrounding liver tissue, with no distinct lesions in the surrounding liver tissue.

HCAs are rare benign neoplasms arising from hepatocytes. Microscopically, tumor cells resemble normal hepatocytes and can have glycogen and lipid degeneration, but they do not show any significant heterogeneity. The tumor cells are arranged in a disordered trabecular structure with a thickness of 1–2 hepatocytes, and pseudo-adenoid structures are occasionally seen. The scattered distribution of thin-walled dilated small veins in the tumor interstitium was one of the important features of HCAs. The tumor had no incomplete tumor capsule, and no chronic hepatitis or cirrhosis was observed in the surrounding liver tissues. Immunohistochemical CD34 staining showed a patchy positive distribution of microvessels because of incomplete capillarization in the tumor tissue. Localized hepatocytes in HCAs can undergo multifocal proliferative alterations, manifested by a larger volume of hepatocytes in the area, lightly stained or translucent cytoplasm covering 1–2 hepatic lobules, capillarization of the hepatic sinusoids, and steatosis of peripheral hepatocytes, which was once called adenomatous hyperplasia. Among HCAs, more than 80% are HHCAs and IHCAs. HHCAs account for 30–40% of HCAs. Histology shows a lobulated outline with steatosis. Liver fatty acid binding protein (L-FABP) expression is negative. L-FABP negative is a marker for HHCAs diagnosis [7]. IHCAs are also known as vasodilatory adenoma, which account for 40–50% of HCAs. The most common pathological type of hepatic adenoma. Histology showed intratumoral hemorrhage, purpura, and sinus telangiectasia, focal or diffuse inflammation. Many thick-walled arteries have bile duct reactions. L-FABP, serum amyloid A (SAA), and C-reactive protein (CRP) are all positive. bHCAs account for 10–15% of HCAs. Based on the histological characteristics, the tumors have a fibrous envelope. Some tumors show pseudoadenoid structures and nodules within a nodule; β-catenin is nuclear positive, glutamine synthetase (GS) diffusive, and strongly positive. This type of HCA is a risk factor for developing HCC [8]. UHCAs account for 10% of HCAs. No characteristic gene mutations, histological features, or immunohistochemical staining features of UHCAs have been found yet. Based on the present data, SAA, CRP, and GS are negative in UHCAs. The occurrence of malignancy in HCAs can be focal; therefore, attention should be paid to multiple points of sampling.

### Differential diagnosis

As a rare pediatric liver tumor, HCAs should be differentiated from the following tumors: (1) Hepatocellular carcinoma (HCC): HCA is difficult to distinguish from well-differentiated HCC. Highly differentiated small HCC is arranged in a thin beam pattern, with widened blood sinusoidal space and distinct pseudo glandular duct structure, and the cancer trabeculae migrate with the paracancerous liver tissue, and a local infiltrative border can appear. Peripheral liver tissue often has chronic hepatitis or cirrhosis. Glypican-3, HSP70, and CD34 immunohistochemical staining were positive in HCC and negative in HCA. (2) Focal nodular hyperplasia of the liver (FNH): FNH is nodular hyperplasia of hepatocytes with central purpura or fibrous septa, often with characteristic bile duct reaction. Immunohistochemical GS showed characteristic “map-like” staining, and CD34-positive microvessels showed characteristic focal distribution. (3) Hepatoblastoma (HB): HB is the most common malignant tumor of the liver in children. HB fetal tumor cells are arranged in cords or sheets, with abundant cytoplasm, and can be red-stained or translucent, mostly with extramedullary hematopoiesis. HCA does not express GPC3, and positive GPC3 can identify benign liver tumors from HB. The nuclear value-added index of HCA is low, and HB accounts for 21.8–44.3% of liver tumors.

### Treatment and prognosis

Along with traditional open surgical resection, laparoscopic hepatectomy, radiofrequency ablation, and transcatheter arterial embolization are available for the treatment of HCAs. For asymptomatic lesions as small as 5 cm in diameter, a conservative treatment plan with close follow-up is possible [9]. Meanwhile, radiofrequency ablation is also an effective treatment for small-diameter HCAs. Mironov et al. [10] treated 36 patients with HCAs with a median tumor size of 2.1 (0.6–6.0) cm by radiofrequency ablation. Postoperative follow-up was 40 months without bleeding or carcinoma of HCA. Asymptomatic HCAs with clinical symptoms and masses larger than 5 cm in diameter should be resected completely as much as possible because incomplete resection can lead to a risk of malignancy and an overall malignancy rate of approximately 4–8% [11]. Malignant transformation is rarely seen in adenomas < 3.5 cm and tumors with prolonged degeneration [12]. Malignant transformation of HCAs is a rare phenomenon in pediatric patients. It has been described in association with GSD type I, FA, CPSS, and Wolff-Hirschhorn syndrome [13]. Rare cases of malignant transformation of HCAs to hepatoblastoma have also been reported. Louie et al. [14] reported hepatoblastoma arising in pigmented bHCAs of a 4-year-old male patient. Ruptured bleeding from HCAs is more commonly reported clinically, and risk factors for bleeding include tumor diameter > 3.5 cm, visible arteries within and around the tumor, adenomas in the left liver lobe, and exophytic adenomas [15]. Death due to HCAs is rare, but almost always due to tumor rupture, abdominal hemorrhage, and shock. Therefore, early surgical treatment should be performed after detection.

## Data Availability

The datasets used and/or analyzed during the current study are available from the corresponding author on reasonable request.

## Abbreviations

HCAs: Hepatocellular adenomas
AFP: α-Fetoprofein
IHC: Immunohistochemistry
FA: Fanconi anemia
GSDs: Glycogen storage diseases
CPSS: Congenital portosystemic shunts
ALP: Alkaline phosphatase
GT: γ-glutamyl transferase
L-FABP: Liver fatty acid binding protein
SAA: serum amyloid A
CRP: C-reactive protein
GS: Glutamine synthetase
FNH: Focal nodular hyperplasia of the liver
HCC: Hepatocellular carcinoma
HB: Hepatoblastoma

## References

[1] Resnick MB, Kozakewich HP, Perez-Atayde AR (1995). Hepatic adenoma in the pediatric age group. Clinicopathological observations and assessment of cell proliferative activity. Am J Surg Pathol.

[2] Hahn E, Putra J (2020). Hepatocellular adenoma in the paediatric population: molecular classification and clinical associations. World J Gastroenterol.

[3] Rooks JB, Ory HW, Ishak KG, Strauss LT, Greenspan JR, Hill AP (1979). Epidemiology of hepatocellular adenoma. The role of oral contraceptive use. JAMA.

[4] Liu HP, Zhao Q, Jin GZ, Qian YW, Gu YJ, Dong H (2015). Unique genetic alterations and clinicopathological features of hepatocellular adenoma in chinese population. Pathol Res Pract.

[5] Zucman-Rossi J, Jeannot E, Nhieu JT, Scoazec JY, Guettier C, Rebouissou S (2006). Genotype-phenotype correlation in hepatocellular adenoma: new classification and relationship with HCC. Hepatology.

[6] Nault JC, Couchy G, Balabaud C, Morcrette G, Caruso S, Blanc JF (2017). Molecular classification of hepatocellular adenoma associates with risk factors, bleeding, and malignant transformation. Gastroenterology.

[7] Margolskee E, Bao F, de Gonzalez AK, Moreira RK, Lagana S, Sireci AN, Sepulveda AR, Remotti H, Lefkowitch JH, Salomao M (2016). Hepatocellular adenoma classification: a comparative evaluation of immunohistochemistry and targeted mutational analysis. Diagn Pathol.

[8] Kwok WY, Hagiwara S, Nishida N, Watanabe T, Sakurai T, Ida H (2017). Malignant transformation of hepatocellular adenoma. Oncology.

[9] Farges O, Ferreira N, Dokmak S, Belghiti J, Bedossa P, Paradis V (2011). Changing trends in malignant transformation of hepatocellular adenoma. Gut.

[10] Mironov O, Jaberi A, Beecroft R, Kachura JR (2018). Retrospective single-arm cohort study of patients with hepatocellular adenomas treated with percutaneous thermal ablation. Cardiovasc Intervent Radiol.

[11] Nault JC, Bioulac-Sage P, Zucman-Rossi J (2013). Hepatocellular benign tumors-from molecular classification to personalized clinical care. Gastroenterology.

[12] Bioulac-Sage P, Sempoux C, Balabaud C (2017). Hepatocellular adenoma: classification, variants and clinical relevance. Semin Diagn Pathol.

[13] Battaglia A, Calhoun ARUL, Lortz A, Carey JC (2018). Risk of hepatic neoplasms in Wolf-Hirschhorn syndrome (4p-): four new cases and review of the literature. Am J Med Genet A.

[14] Louie CY, Concepcion W, Park JK, Rangaswami A, Finegold MJ, Hazard FK (2016). Hepatoblastoma arising in a pigmented β-catenin-activated Hepatocellular Adenoma: case report and review of the literature. Am J Surg Pathol.

[15] Julien C, Le Bail B, Balabaud C, Bioulac-Sage P (2022). Risk factors for bleeding hepatocellular adenoma. Liver Int.

